# Genome-scale NCRNA homology search using a Hamming distance-based filtration strategy

**DOI:** 10.1186/1471-2105-13-S3-S12

**Published:** 2012-03-21

**Authors:** Yanni Sun, Osama Aljawad, Jikai Lei, Alex Liu

**Affiliations:** 1 Department of Computer Science and Engineering, Michigan State University, East Lansing, MI 48824, USA

## Abstract

**Background:**

NCRNAs (noncoding RNAs) play important roles in many biological processes. Existing genome-scale ncRNA search tools identify ncRNAs in local sequence alignments generated by conventional sequence comparison methods. However, some types of ncRNA lack strong sequence conservation and tend to be missed or mis-aligned by conventional sequence comparison.

**Results:**

In this paper, we propose an ncRNA identification framework that is complementary to existing sequence comparison tools. By integrating a filtration step based on Hamming distance and ncRNA alignment programs such as FOLDALIGN or PLAST-ncRNA, the proposed ncRNA search framework can identify ncRNAs that lack strong sequence conservation. In addition, as the ratio of transition and transversion mutation is often used as a discriminative feature for functional ncRNA identification, we incorporate this feature into the filtration step using a coding strategy. We apply *Hamming distance seeds *to ncRNA search in the intergenic regions of human and mouse genomes and between the *Burkholderia cenocepacia *J2315 genome and the *Ralstonia solanacearum *genome. The experimental results demonstrate that a carefully designed Hamming distance seed can achieve better sensitivity in searching for poorly conserved ncRNAs than conventional sequence comparison tools.

**Conclusions:**

Hamming distance seeds provide better sensitivity as a filtration strategy for genome-wide ncRNA homology search than the existing seeding strategies used in BLAST-like tools. By combining Hamming distance seeds matching and ncRNA alignment, we are able to find ncRNAs with sequence similarities below 60%.

## Introduction

Identifying ncRNAs (non-coding RNAs), which function directly as RNAs rather than being translated into proteins, has drawn tremendous attention recently for two main reasons. First, besides well-known functions in protein-synthesis, regulatory roles of small ncRNAs have been revealed in gene regulation [1] in a wide variety of species. Second, new members of annotated ncRNA families or novel ncRNAs have been identified due to advances of the next-generation sequencing technologies and RNA-seq. Understanding ncRNAs plays a key role in elucidating the complexity of regulatory network of both complicated and simple organisms.

**Figure 1:**
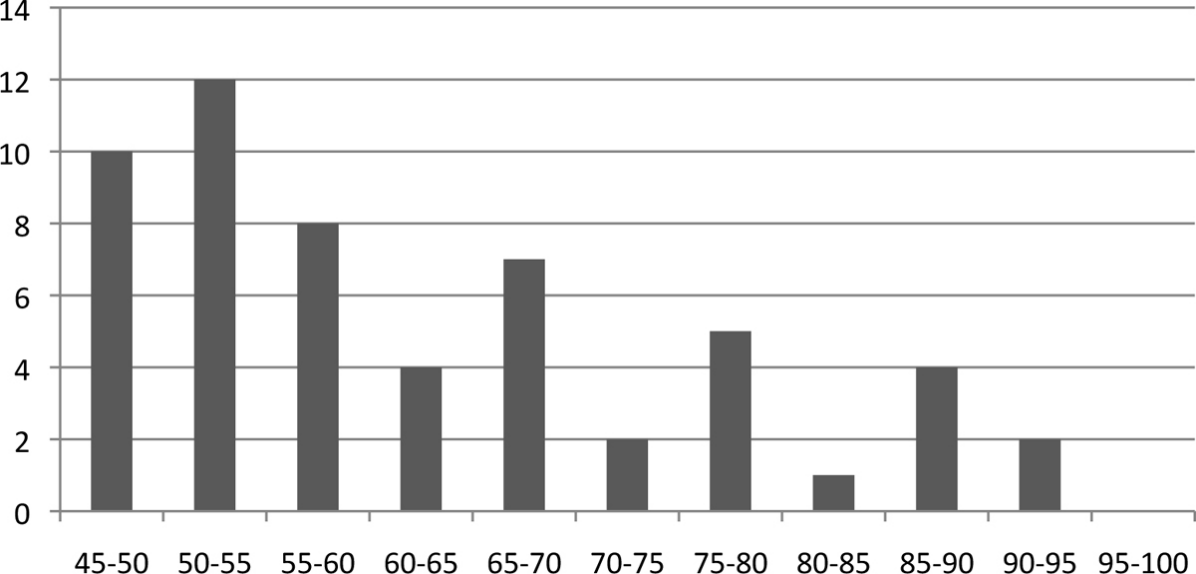
**The sequence similarity histogram of homologous tRNAs in the human and mouse genomes.** The X-axis is the sequence similarity. The Y-axis is the number of homologous tRNA pairs. The sequences are obtained from the tRNA family in the Rfam [8] database. For each human tRNA, we report the highest sequence similarity.

The state-of-the-art methodology for ncRNA annotation is based on comparative analysis, which searches for evolutionarily conserved ncRNAs in related genomes or their transcriptomes. Existing genome-scale ncRNA identification methods [2-4] first employ conventional sequence comparison tools such as BLAST [5] to generate an initial set of alignments for further screening. Then, features such as secondary structure conservation, minimum free energy (MFE), sequence conservation, GC content, base or basepair substitution patterns etc. [3,6] are employed to classify these local alignments as putative ncRNAs, protein-coding genes, or other genomic features. However, although BLAST-like sequence comparison tools have been successfully used for finding protein-coding genes, segment duplications, and other genomic features, they are not well suited for comprehensive ncRNA search. NCRNAs function through both their sequences and structures. Some types of ncRNA evolve faster in their sequences than in their secondary structures and thus have low sequence conservation. For example, RNase P is highly structured and cannot be found by conventional sequence similarity search tools [1]. Many lineage specific ncRNAs such as Xist or Air have very low sequence conservation [7] and pose hard cases for BLAST-like tools. Even some small ncRNAs such as tRNA have a wide range of sequence conservation. Figure 1 shows the histogram of sequence similarity between homologous tRNAs in the human and mouse genomes. More than half of the homologous tRNAs have similarity below 60%.

**Figure 2:**
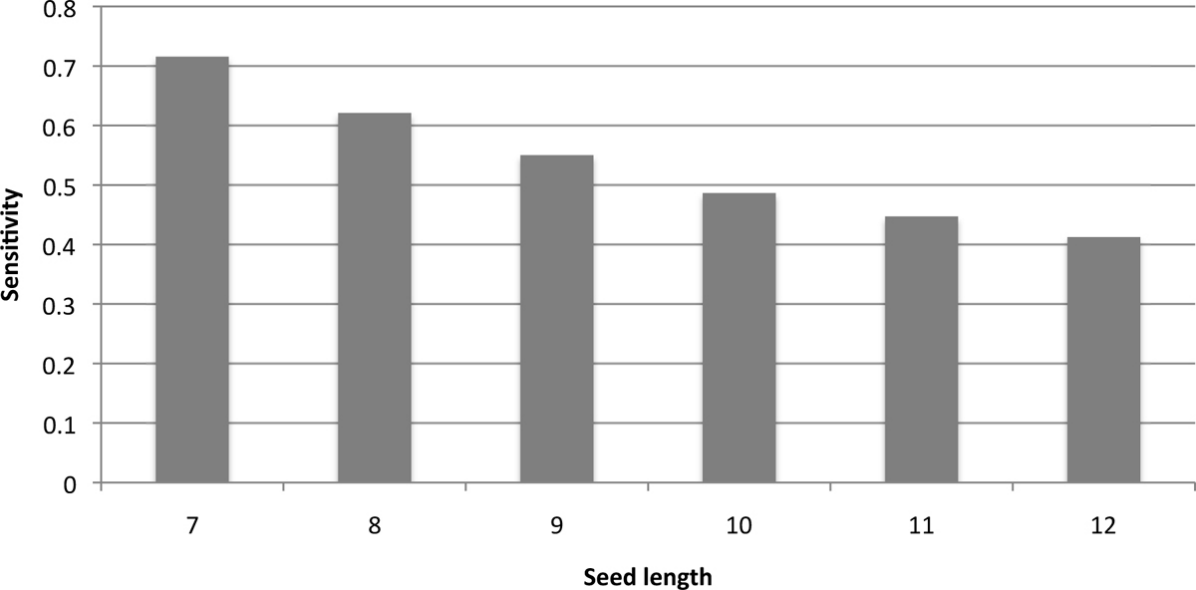
**The sensitivity of the BLAST seeds of different lengths on 3925 homologous ncRNAs between human and mouse genomes.** X-axis is the length of seeds. Y-axis is the sensitivity.

BLAST-like sequence comparison tools tend to miss these ncRNAs for two reasons. First, genome-scale sequence comparison tools use the seed-and-extend scheme, where efficient exact matching of short patterns (i.e. seeds) is used as the filtration step to locate regions that are likely to be true homologs. Full dynamic programming is only applied to regions around seed hits. However, as the sequence similarity decreases, the probability that homologous regions contain a match to the seed also decreases fast. As a result, these ncRNAs will be missed in the filtration step. In order to quantify how the seeding heuristic in BLAST affects ncRNA homology search, we extracted 3925 pairs of homologous ncRNAs from the human and mouse genomes from Rfam 10 [8]. For each pair of homologous ncRNAs, we test whether they match a seed of different length. The result is summarized in Figure 2. When we use the default seed size 11 in BLAST, there are only 1755 (i.e. 45%) pairs of ncRNAs passing the filtration step. Although spaced seeds [9-11] have been used to improve BLAST's sensitivity, ncRNAs lack sequence signatures or characteristics such as the triplet amino acid code for protein coding gene detection, posing great challenges for seed design. We tested the spaced seed in BLASTZ on the same data set. The sensitivity is **0.517**. BLASTZ adopts the optimal spaced seed (1110100110010101111) designed by PattermHunter [9], but allows a transition mutation in one of matching positions (i.e. positions with 1) in order to improve the tradeoff between seed detection sensitivity and false positive rate.

The second problem of using BLAST-like tools for ncRNA identification is that they do not incorporate structural similarity. Deriving secondary structure on pure sequence alignment has limited accuracy. Previous work [12] has shown that the final alignments generated by BLAST and structural alignment tools such as FOLDALIGN [13,14] can be quite different.

In order to conduct ncRNA search efficiently and accurately, we propose a new approach that integrates a sensitive filtration step with a local ncRNA alignment step for identifying homologous ncRNAs. The filtration step locates substrings with Hamming distance smaller than a given threshold. By carefully choosing the length and distance threshold for Hamming distance, we can locate all regions within a range of sequence similarity. In the second step, the regions passing the filtration stage will be used as input to ncRNA alignment programs, which are designed to incorporate both the sequence and structural similarities in ncRNAs. There are a number of ncRNA alignment tools available. As output of the filtration stage does not indicate the exact starting or ending positions of putative ncRNAs, local alignment tools are desired. In this work, we used two types of ncRNA alignment programs for the second stage and compared their performance. The two types of programs are based on different methodologies. One folds and aligns sequences simultaneously to maximize both sequence and structural similarity. The other uses posterior probability alignment to boost homology search sensitivity. NcRNAs that may be missed by conventional sequence comparison tools have higher probability to be identified using these alignment programs.

We applied this approach to ncRNA homology search between intergenic regions in human and mouse genomes [15], and between the *Burkholderia cenocepacia *J2315 genome and the *Ralstonia solanacearum *genome [16]. The experimental results demonstrate that our approach is efficient and is more sensitive than conventional sequence alignment tools for finding ncRNAs with sequence identity below 60%.

## Related work

There are a number of ncRNA alignment tools that incorporate both sequence and structural similarities. However, most of them are based on global alignment, requiring known starting and ending positions of ncRNAs. Identifying ncRNAs in genomes or transcriptome data sets requires local ncRNA alignment. FOLDALIGN is a highly sensitive local structural alignment tool that can identify ncRNAs with very low sequence similarity (*<*40%). Using heuristics such as dynamic programming matrix pruning, FOLDALIGN is faster than the accurate implementation of the Sankoff algorithm [17]. However, it is still CPU-intensive on large data sets. When it is applied for ncRNA search between the intergenic regions of the human and mouse genomes, FOLDALIGN took about 5 months on 70 2-GB-RAM nodes in a linux cluster [15]. Thus, it is not practical to directly apply FOLDALIGN to large sequence sets.

Because of the cost of structural alignment, existing genome-scale ncRNA search tools [2-4] still rely on conventional sequence alignment programs such as BLAST. As one of seeded alignment tools, BLAST relies on its seeding heuristics to achieve efficiency of local similarity search between long genomes. Both the theoretical analysis and empirical experiments [9,18] have shown that choice of the seeding heuristics affects the sensitivity of local alignments. While BLAST requires consecutive matching, PatternHunter [9] allows spaced seeds, which can incorporate biological features of the underlying alignments. For example, spaced seeds designed for coding regions allow a mismatch following two exact matches, indicating the less strictly specified base in a codon. However, it is much more difficult to design useful spaced seeds for ncRNA search because 1) ncRNAs do not preserve strong sequence characteristics; 2) we lack enough training sequences for seed design. A more advanced seed type than spaced seed distinguishes transition and transversion as many functional genomic features including ncRNAs show a higher frequency of transition than transversion [18-20]. This type of seed is adopted by sequence comparison tool BLASTZ [19]. It uses the optimal spaced seed designed by PatternHunter but allows a transition mutation (A-G, G-A, C-T, or T-C) at any one of the inspected positions in the seed.

Recently, a posterior-probability based ncRNA local alignment tool PLAST-ncRNA has been implemented [21]. However, it is designed to align a relative short query sequence with a long target sequence rather than between two genomes. Thus, it cannot be directly applied to genome-scale ncRNA search without manually dividing a long genome into numerous small segments.

In our work, we design a filtration strategy based on Hamming distance. There are a number of existing implementations that search for substrings satisfying a pre-defined Hamming distance threshold. For example, in the ungapped short read mapping problem, short reads generated from next-generation sequencing platforms are aligned to the reference genome by allowing a couple of mismatches. Techniques such as neighborhood generation and the pigeon hole theory have been applied to transform inexact match to exact match in order to improve the search speed. Although a number of efficient read mapping programs [22,23] exist, they cannot be used as the filtration step in ncRNA search because read mapping usually only allows a very small number of mismatches. In addition, they are specifically designed to align a set of short reads with a long reference genome.

## Methods

**Figure 3:**
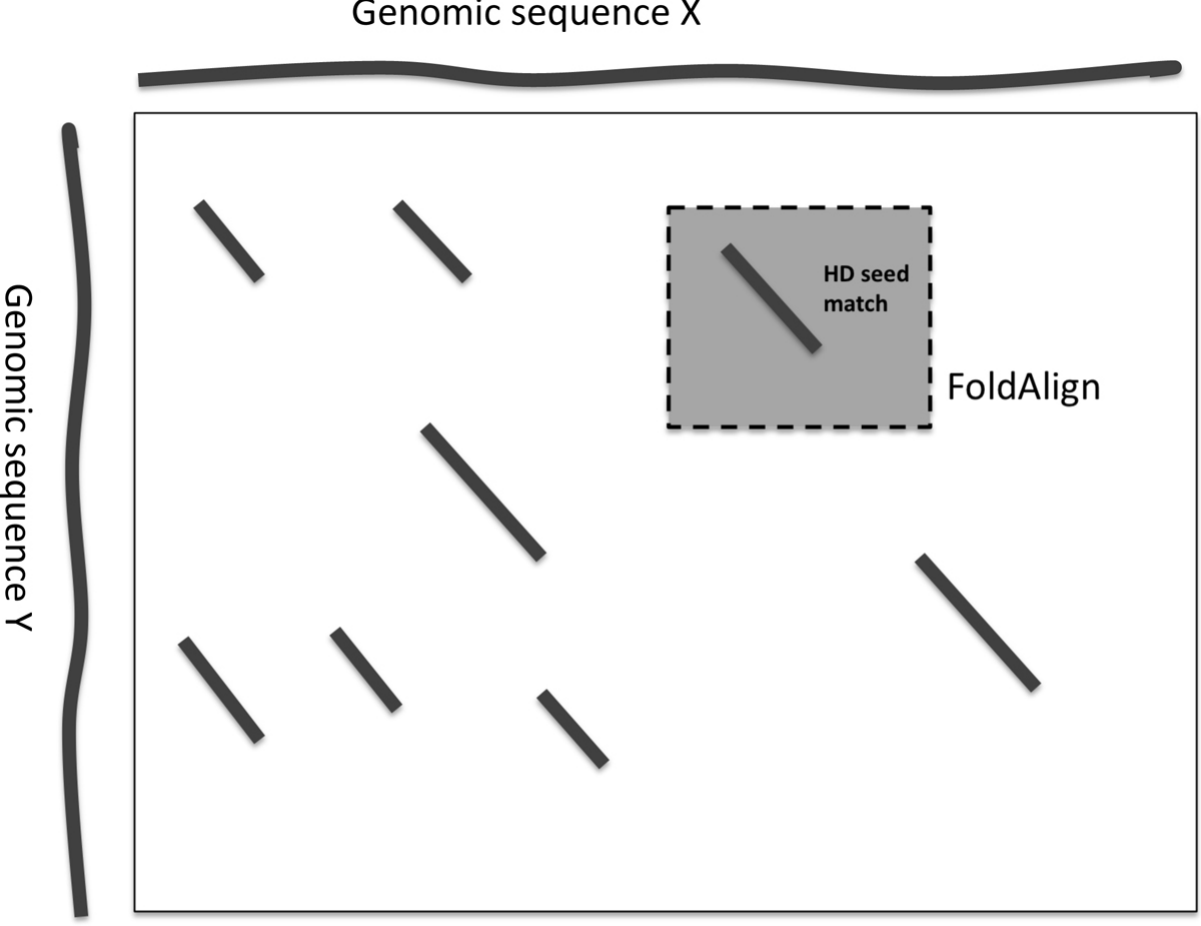
**The framework of genome-scale ncRNA search using HD seeds.** In the first step, HD seed hits (represented by diagonal lines) are identified. Then more sensitive local structural alignment tools such as FOLDALIGN and PLAST-ncRNA are applied in the region surrounding a seed hit. Subsequent analyisis can be conducted on the output of the second stage.

Hamming distance is the number of mismatches in two strings of equal length. Based on Hamming distance, we define *HD seeds *(Hamming distance seeds) as a 2-tuple *<*L, T*>*, where L is the length of the seed and T is the threshold. A Hamming seed *<*L, T*> matches *a pair of strings of equal length L if the Hamming distance between two inputs is equal to or less than *T*. According to the definition of Hamming distance, any pair of input strings of length L with sequence similarity at least $\frac{L-T}{L}$ can be matched by the HD seed *<*L,T*>*. Thus, by choosing appropriate L and T, we can use HD seed matching as the filtration step to locate possible ncRNAs with low sequence conservation. Then we extend the seed hit to both directions and apply a local structural alignment method in the vicinity of the seed hit for more sensitive ncRNA screening. The pipeline of this method is illustrated in Figure 3.

In the remaining part of this section, we first describe the coding system that can distinguish transition from transversion in Hamming distance seeds. Then we present optimal HD seed generation.

### Design a coding system to distinguish transition from transversion

Transition mutations are less likely to result in amino acid changes. Thus, it is expected that transitions are observed at higher frequency than transversions in homologous protein-coding genes. This fact has been adopted by sequence alignment tools such as BLASTZ to improve the performance of homology search. Similar observations have been made in homologous ncRNAs as well. In the score table RIBOSUM designed by Klein and Eddy [24], transitions in both single stranded regions and between base pairs have higher scores than transversions. Higgs [20] reported that the substitution rate between a base pair (such as AU) and its double transition base pair (such as GC) is significantly higher than other mutations. Thus, it is desirable to distinguish transition from transversion in our HD seeds. However, the Hamming distance defined on DNA or RNA bases treats each mismatch equally. In order to favor transition over transversion in HD seeds, we formulate the following coding problem.

First, all bases are encoded by binary strings of equal length. Let the length be *s*. For each base x, let x.code denote the encoded binary string. Let the function D(x,y) be the hamming distance of x.code and y.code, where × and y are two bases. For bases A, C, G, T, we need to determine their codes such that the following equations are satisfied:

(1) $$\begin{array}{llll} D\left(A,\;G\right) & ==D\left(C,\;T\right);\; & & \;\\ D\left(A,\;C\right) & >D\left(A,\;G\right);\; & & \;\\ D\left(A,\;C\right) & ==D\left(A,\;T\right)\; & & \;\\ & ==D\left(C,\;G\right)\; & & \;\\ & ==D\left(G,\;T\right);\; & & \;\\ & \; & \end{array}$$

Multiple codes exist. The shortest codes for the above problem are presented in Table 1. In the coded binary strings, the distance of exact match is zero; the distance for transition is 2; the distance for transversion is 3. As a result, the Hamming distance not only depends on the number of substitutions in a pair of input strings, but also the ratio of transition to transversion. For example, string "CCCCC" has Hamming distance 3 with both "CUCUU" and "CGCGG". After encoding, the corresponding bit strings have Hamming distances 6 and 9, respectively. Generally speaking, for two genomic sequences with equal length, if there are *x*_1 _matches, *x*_2 _transitions, and *x*_3 _transversions, the HD distance is 2*x*_2 _+ 3*x*_3 _on two binary strings with length 4 *× *(*x*_1 _+ *x*_2 _+ *x*_3_).

**Table 1:** Converting bases into bits

Base	Binary codes
A	1111
C	0001
G	1100
T(U)	0010

### Hamming distance seed design

To design an HD seed, we need to determine L and T to maximize its matching probability in ncRNA homologs while keeping the matching probability to random sequences as low as possible. Given a pair of true ncRNA homologs, the probability that the input pair contains a match to the given HD seed is proportional to the sensitivity of the seed. Given a pair of random sequences, the probability that the input pair contains a match to the given seed is proportional to the false positive (FP) rate of the seed. Thus, computing the matching probability allows us to compare performance of different seeds. As there are a large number of valid combinations of L and T, an efficient method is needed for the matching probability computation. In this work, we use a simple i.i.d. model to describe distributions of exact matches, transitions, and transversions in a pair of sequences. The theoretical HD seed matching probability can be efficiently computed based on the i.i.d. model.

The i.i.d. model M is defined as a 3-tuple *<p*_1_, *p*_2_, *p*_3_*>*, where *p*_1_, *p*_2_, and *p*_3 _are the probabilities of exact match, transition, and transversion, respectively. Thus, *p*_1 _+ *p*_2 _+ *p*_3 _= 1.0. In order to compute the matching probability of an HD seed *<*L,T*>*, we start with the probability that a pair of sequences of length *l *contain *x*_1 _exact matches, *x*_2 _transitions, and *x*_3 _transversions as follows:

(2) $$\begin{array}{llll} Pr^{M}\left(x_{1},\;x_{2},\;x_{3}\right) & =\left(\begin{array}{c} l\\ x_{1} \end{array}\right)p_{1}^{x_{1}}\left(\begin{array}{c} l-x_{1}\\ x_{2} \end{array}\right)p_{2}^{x_{2}}p_{3}^{x_{3}}\; & & \;\\ & =\frac{l!}{x_{1}!x_{2}!x_{3}!}p_{1}^{x_{1}}p_{2}^{x_{2}}p_{3}^{x_{3}}\; & & \;\\ & \; & \end{array}$$

where *l *= *x*_1 _+ *x*_2 _+ *x*_3_. As we convert bases into binary codes according to rules in Table 1 before applying HD seed matching, the matching probability of an HD seed *<*L, T*>*can be represented using $Pr^{M}\left(x_{1},\;x_{2},\;x_{3}\right)$ as below:

(3) $$Pr^{M}\left(L,\;T\right)=\underset{x_{1}+x_{2}+x_{3}=L/4;\;2*x_{2}+3*x_{3}\leq T}{\sum }Pr^{M}\left(x_{1},\;x_{2},\;x_{3}\right)$$

For an HD seed *<*L,T*>*, there are multiple combinations of *x*_1_, *x*_2_, and *x*_3 _satisfying the above equation. The matching probability must sum over all combinations. In the above equations, *l *is the number of bases in genomic sequences and *L *is the number of bits after coding.

**Figure 4:**
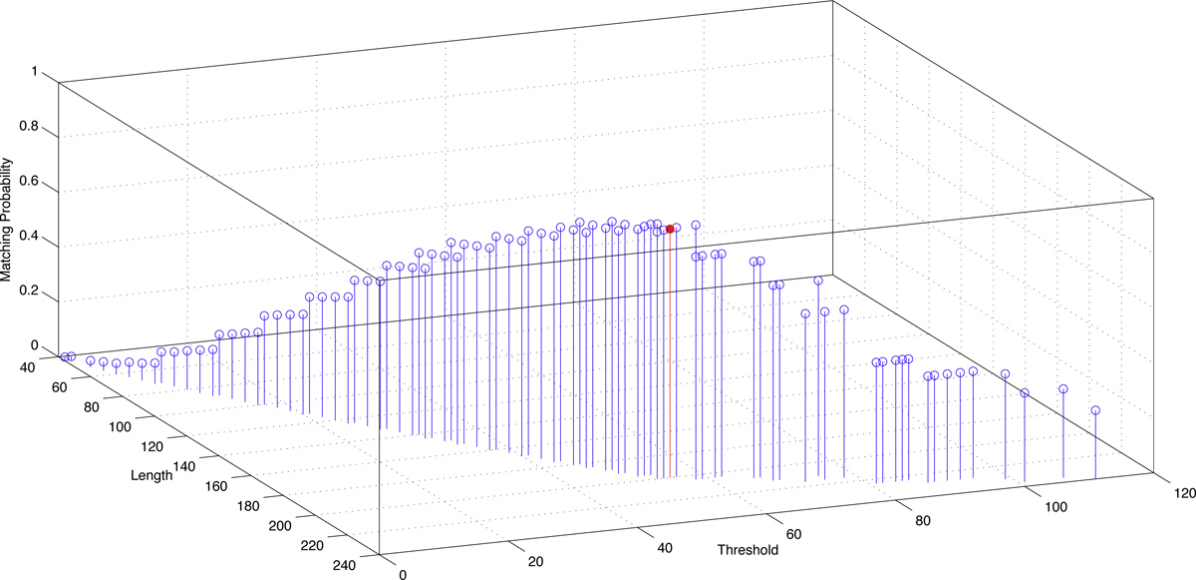
**Matching probabilities of HD seeds of different length L and threshold T in true ncRNA homologs.** To make points distinguishable, a large number of seeds with matching probability close to zero (i.e. low sensitivity) are not shown.

**Figure 5:**
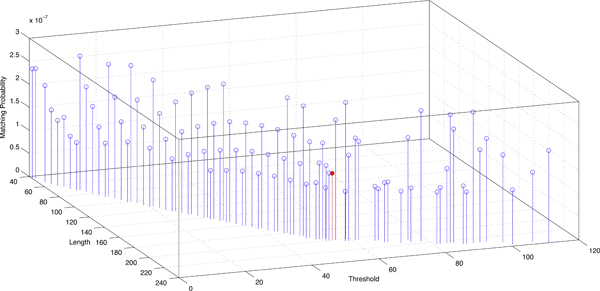
**Matching probabilities of HD seeds of different length L and threshold T in random sequences.** To make points distinguishable, a large number of seeds with matching probability close to 1 (i.e. high FP rate) are not shown.

The choice of L and T heavily depends on probabilities of matching and transition in M. To compute matching probabilities in true ncRNA homologs, we train M on pairwise ncRNA alignments from seed families in Rfam version 10. M=*<*0.683, 0.154, 0.163*>*. In order to compute HD seed matching probability in random sequences, which indicates the false positive rate, we assume that the four bases occur with the same probability. Thus, in the i.i.d. model $M^{\prime }$, *p*_1 _= 0.25, *p*_2 _= 0.25, and *p*_3 _= 0.5. By applying M and $M^{\prime }$ to Eqn. 3, we can use values of $Pr^{M}\left(L,\;T\right)$ and $Pr^{M^{\prime }}\left(L,\;T\right)$ to quantify the performance of HD seeds with different length and threshold. There are total 5551 different HD seeds with length smaller than 60 bases (i.e. 240 bits). After removing seeds which can incur FP rate near 1 or sensitivity near 0, we plot $Pr^{M}\left(L,\;T\right)$ and $Pr^{M^{\prime }}\left(L,\;T\right)$ for the remaining seeds in Figures 4 and 5. These two figures illustrate how the seed length and threshold affect the seed's matching probabilities.

Based on the two figures, we determine L and T with the best tradeoff between $Pr^{M}\left(L,\;T\right)$ and $Pr^{M^{\prime }}\left(L,\;T\right)$. The chosen seed is *<*200,55*>*, which is highlighted in Figures 4 and 5. Its matching probability in true ncRNA homologs is 0.906 and its matching probability in random sequences is 1.45E-07. The seed *<*200,55*>*represents a similarity $\frac{200-55}{200}=72.5\%$ on coded bit strings. According to the coding Table 1, for genomic sequence of length 50 = 200*/*4, the seed *<*200,55*>*allows 26 transition and 1 transversion mutation. This combination gives the lowest DNA-level similarity 46% = (50 *- *26 *- *1)*/*50. Thus, this chosen seed is able to detect highly structured ncRNAs which have very low sequence conservation.

### Softwares for HD seed matching and local structural alignment

There are a number of tools that can implement HD seed matching. We chose a randomized algorithm LSH-ALL-PAIRS [25], which is based on locality sensitivity hashing. Although it is an approximation algorithm, it has achieved high sensitivity in detecting DNA homologs with similarity as low as 63%. More importantly, it is fast enough to apply to whole genomes even when the allowed substitutions (i.e. T in the HD seeds) increases.

For a pair of substrings that contain a match to the HD seed, we apply two types of local alignment programs. The first is FOLDALIGN, which can conduct local structural alignment. The second is PLAST-ncRNA, which uses posterior probabilities to conduct alignments. Both of these tools can detect homologous ncRNAs with low sequence similarities.

LSH-ALL-PAIRS, FOLDALIGN, and PLAST-ncRNA were downloaded from the authors' websites.

### Experiments and results

For ncRNAs with high sequence similarity, BLAST and other seeded alignment tools suffice to identify them between related genomes. The goal of our tool is to provide complementary ncRNA identification method to conventional sequence comparison tools. In this section, we focus on testing ncRNA search performance of HD seeds in data sets with low sequence conservation.

The focus of the first experiment is to search for putative structural ncRNAs in genomic regions in human that could not be aligned with mouse. Torarinsson et al. [15] directly applied FOLDALIGN for ncRNA search in a set of intergenic regions in the two genomes. Structural ncRNAs with high confidence are revealed. From the website of the paper, we downloaded 1297 alignments, which have high probabilities to be functional ncRNAs. These ncRNA pairs have low sequence similarities (48% on average) and a majority of them cannot be aligned by BLAST. We apply BLAST, BlastZ, and Hamming seeds to this data set and quantify their *sensitivity *and *FP rate *(false positive rate). Sensitivity evaluates the percentage of true homologs (i.e., 1297 alignments) that can be aligned by these programs. FP rate evaluates how many pairs of random sequences can be aligned by these programs. In order to compute the FP rate, we generated 10,000 pairs of random sequences assuming each base has the same probability. The sensitivity and FP rate are summarized in Table 2. According to Table 2, HD seed has the best sensitivity and also low FP rate. BlastZ has the higher sensitivity than BLAST. This experiment shows that using HD seeds to locate possible ncRNA homologs is more sensitive than using conventional sequence comparison programs.

**Table 2:** Comparison of Hamming seeds, BLAST, and blastZ

	HD seed	BLAST	BlastZ
Sensitivity	**0.6**	0.07	0.17
FP rate	**0.0009**	0.0011	0.0054

### NcRNA search in the Burkholderia cenocepacia J2315 genome

In the second experiment we focus on ncRNA identification in the *Burkholderia cenocepacia *J2315 genome by comparing it with the *Ralstonia solanacearum *genome. *Burkholderia cenocepacia *is clinically important because it can cause lung infections in cystic fibrosis (CF) patients [16]. There are multiple members in *Burkholderia cenocepacia*. Coenye et al. conducted ncRNA search by applying BLAST and QRNA between *B. cenocepacia *strain J2315 and related genomes including the *Ralstonia solanacearum *genome. As BLAST can miss highly structured ncRNAs, we conducted a complementary analysis using HD seeds and ncRNA alignment programs including FOLDALIGN and PLAST-ncRNA. We applied both tools to regions around HD seed hits and compared the outputs of FOLDALIGN and PLAST-ncRNA. We downloaded the three chromosomes (accession IDs: NC_011000, NC_011001, NC 011002) of the *Burkholderia cenocepacia *J2315 genome from NCBI. Their sizes are 3,870,082 nt, 3,217,062 nt, and 875,977 nt, respectively. Similarly we downloaded the *Ralstonia solanacearum *GMI1000 genome (NC_003295) from NCBI. The single chromosome has length 3,716,413 nt. Using BLAST and QRNA, Coenye et al. [16] reported 78, 116, and 19 putative ncRNAs on the three chromosomes of J2315.

We first masked all low-complexity repeats and annotated protein-coding genes in input sequences. Then we applied our designed HD seed *<*200,55*>*between the three chromosomes of *Burkholderia cenocepacia *J2315 and the genome of *Ralstonia solanacearum*. Between every pair of input sequences, the total number of possible matching positions is bounded by the product of the input sequences' sizes. For example, for a seed of size 50 bases, there could be at most (3, 870, 082 *- *49) *× *(3, 716, 413 *- *49) distinct seed matching places. Thus, in general, when the sizes of input sequences increase, more seed hits are expected. The total number of seed hits and the ones that overlap with reported putative ncRNAs by Coenye et al. are summarized in Table 3. Our HD seed detected all putative ncRNAs on chromosome 1 and 3. The HD seed missed 10 putative ncRNAs on chromosome 2 because they are either masked as low-complexity repeats or heavily overlap with annotated coding regions. Thus the corresponding regions are masked and will not be scanned by the HD seed. Previous literature [15] on ncRNA search suggests that most ncRNAs are in intergenic regions in bacterial genomes. It needs extensive investigation whether ncRNA genes overlap protein coding genes in bacterial genomes.

**Table 3:** Comparison of the HD seed hits with putative ncRNAs reported by Coenye et al.

	Putative	HD seed	Overlapped
	ncRNAs	hits	
Chr1	78	162311	78
Chr2	116	14336	106
Chr3	19	2740	19

As the purpose of this experiment is to identify highly structural ncRNAs that might be missed by existing ncRNA homology search tools such as the combination of BLAST and QRNA, we are only interested in seed hits with identity no more than 60%. For each intergenic seed hit with identity no more than 60%, we extended it to left and right for 100 bases in each input. Then local alignment was conducted between extended substrings using FOLDALIGN or PLAST-ncRNA. As chromosome 2 and chromosome 3 are much larger than chromosome 3 and may have more putative ncRNAs, we only present results of search on chromosome 1 and chromosome 2. All programs run on a 128-node cluster, where each node contains 2 dual-core AMD Opterons running at 2.2 GHz with 8 GB of memory. The running time of HD seed matching using LSH-ALL-PAIRS is 8,250 and 6,850 seconds for chromosome 1 and chromosome 2, respectively. The running times of FOLDALIGN on regions around seed matches are 15 hours and 14 hours for chromosome 1 and chromosome 2, respectively. The running times of PLAST-ncRNA on regions around seed matches on chromosome 1 and chromosome 2 are 697 seconds and 501 seconds, respectively. As FOLDALIGN is based on a computationally intensive structural alignment algorithm by Sankoff [17], it takes a much longer running time than posterior-probability based PLAST-ncRNA. However, FOLDALIGN can output both the alignment and the consensus secondary structure for each input pair while PLAST-ncRNA does not provide secondary structure derivation. Additional ncRNA structure prediction programs are needed to process the output of PLAST-ncRNA when structure information is needed.

For all output alignments by FOLDALIGN and PLAST-ncRNA, we remove an alignment if it satisfies one of the following conditions: 1) the alignment overlaps with adjacent protein-coding genes; 2) the alignment score is smaller than a given cutoff; and 3) the alignment length is smaller than 55. PLAST-ncRNA has a cutoff for average posterior probability, which is the normalized posterior probability over the length of an alignment. The default cutoff for PLAST-ncRNA is 0.1. There is no default score cutoff for FOLDALIGN when we conduct the alignment using "local" mode. The "scan" mode provides p-values, which interpret the significance of alignment scores in a better way than the raw scores. Following the assumption made by FOLDALIGN that the alignment scores follow an extreme-value distribution, we designed a score cutoff corresponding to the p-value of 10*^-^*^8^. Specifically, we generated 50,000 random sequences of length 200 and aligned all pairs of them. Then we conducted curve-fitting using the random alignment scores and determined the score cutoff for the chosen p-value. The computed score cutoff for FOLDALIGN is 450.

**Figure 6:**
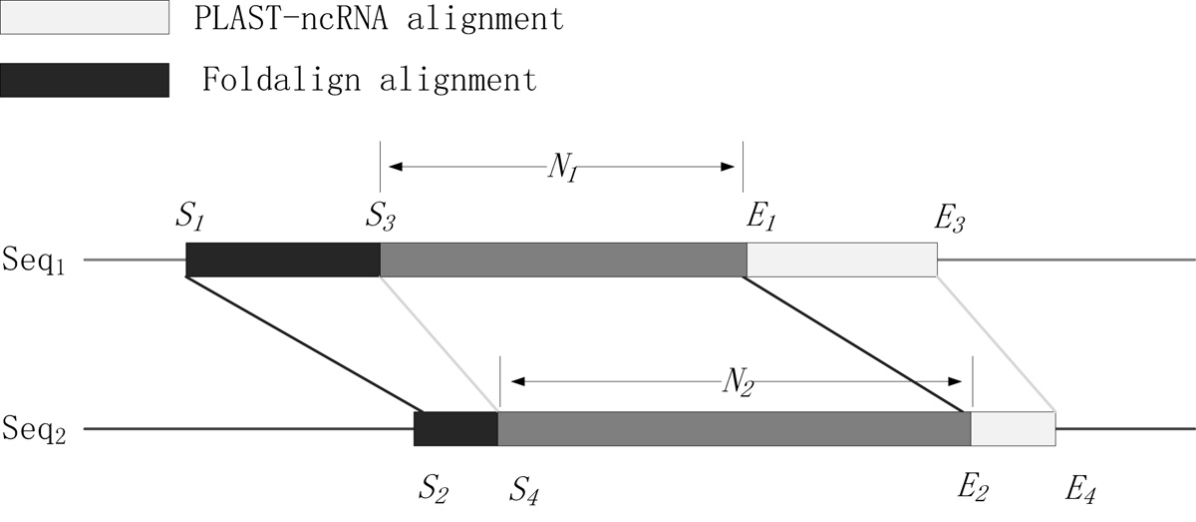
**Definition of overlapping alignments.** Two alignments are output by FOLDALIGN and PLAST-ncRNA. The FOLDALIGN alignment is located between positions *S*_1 _and *E*_1 _on *Seq*_1 _and between *S*_2 _and *E*_2 _on *Seq*_2_. The PLAST-ncRNA alignment is located between positions *S*_3 _and *E*_3 _on *Seq*_1 _and between *S*_4 _and *E*_4 _on *Seq*_2_. *N*_1 _is the overlapping region on *Seq*_1 _and *N*_2 _is the overlapping region on *Seq*_2_.

Based on the above filtration criteria, we kept 8,112 and 6,506 FOLDALIGN alignments on chromosome 1 and 2, respectively. For PLAST-ncRNA under the default cutoff 0.1, we kept 9,263 and 7,233 alignments on chromosome 1 and 2, respectively. By comparing their alignment positions, we found that there is a large overlap between the two sets of output alignments by FOLDALIGN and PLAST-ncRNA. Figure 6 illustrates our definition of overlapping alignments. Given two alignments defined by their starting and ending positions, we calculate the overlapping percentage on each input sequence. Following the notations for the example alignment in Figure 6, the overlapping percentage on the sequence *seq*_1 _is $\frac{N_{1}}{\text{min}\left(\left(E_{1}-S_{1}+1\right),\left(E_{3}-S_{3}+1\right)\right)}$. Similarly, the overlapping percentage on the sequence *seq*_2 _is $\frac{N_{2}}{\text{min}\left(\left(E_{2}-S_{2}+1\right),\left(E_{4}-S_{4}+1\right)\right)}$. Two alignments overlap if the overlapping percentages on both sequences are at least 50%. According to this overlapping alignment criterion, 7,910 and 6,346 alignments are shared by FOLDALIGN and PLAST-ncRNA for chromosome 1 and 2, respectively. Although FOLDALIGN and PLAST-ncRNA are implemented based on highly different methodologies, they give consistent evidence for ncRNA search. As PLAST-ncRNA is near two orders of magnitude faster than FOLDALIGN, we conduct a closer examination of the output of PLAST-ncRNA.

**Figure 7:**
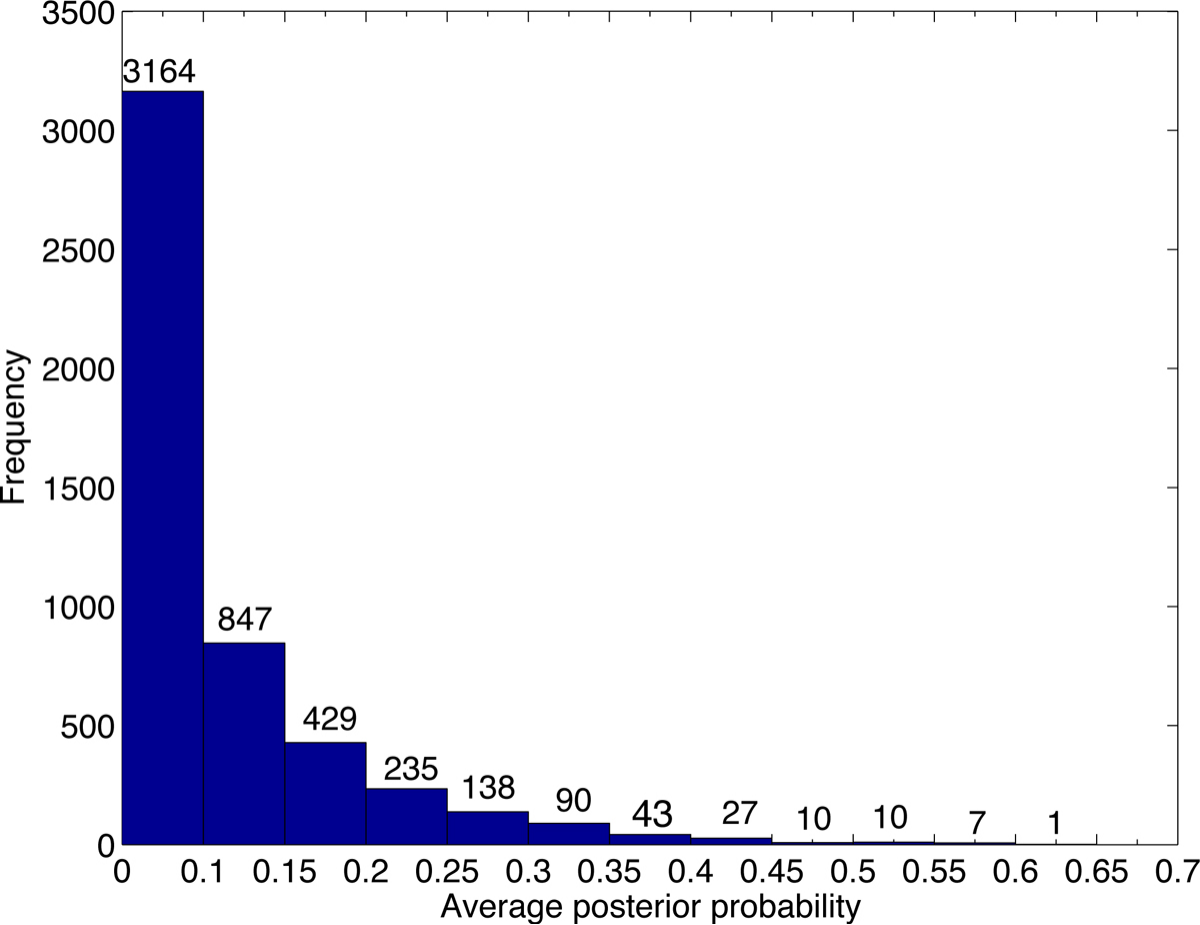
**Average posterior probability distribution for random PLAST-ncRNA alignments.** For each bar between labels × and y, it contains all alignments with average posterior probability *≥ × *and *< y*. The number of alignments for each bar is shown above the bar.

**Figure 8:**
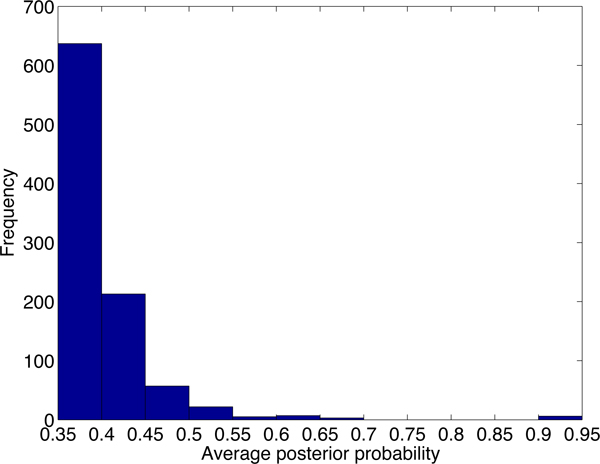
The average posterior probability distribution of PLAST-ncRNA alignments on chromosome 1.

Although there are thousands of alignments passing the default cutoff of PLAST-ncRNA, it is not likely that all of the alignments contain functional ncRNAs. We first examine the default cutoff by generating posterior probability distributions for PLAST-ncRNA alignments for random sequences and known ncRNAs with low sequence similarities. Figure 7 plots the distribution of average posterior probabilities for alignments on 5,000 random sequences of lengths between 60 and 70. There are 37% of alignments with average posterior probability above 0.1, indicating that the default cutoff 1.0 can incur high false positive rate for ncRNA search. As we are only interested in ncRNA homologs with low sequence similarities, we also examine the PLAST-ncRNA probabilities for tRNA and SECIS homologs between human and mouse because these two have low sequence conservations. The minimum average posterior probability is 0.35. Thus, instead of using 0.1, we chose 0.35 as the cutoff for ncRNA search in this experiment. By using the more stringent cutoff, PLAST-ncRNA output 954 and 716 alignments on chromosome 1 and 2, respectively. For these alignments, we plot their average posterior probabilities, sequence identity, and alignment length in the figures from Figure 8 to Figure 13.

**Figure 9:**
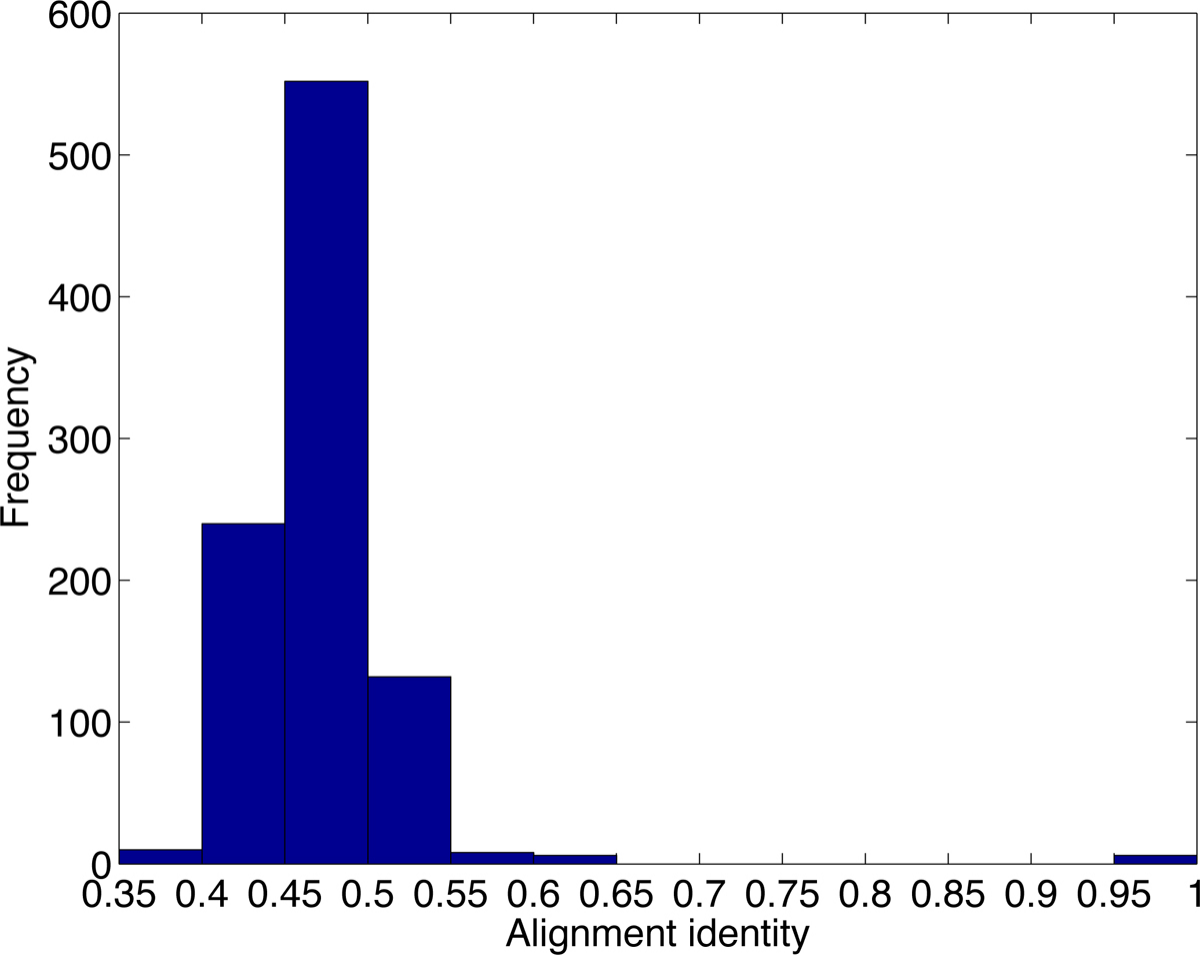
The sequence identity distribution of PLAST-ncRNA alignments on chromosome 1.

**Figure 10:**
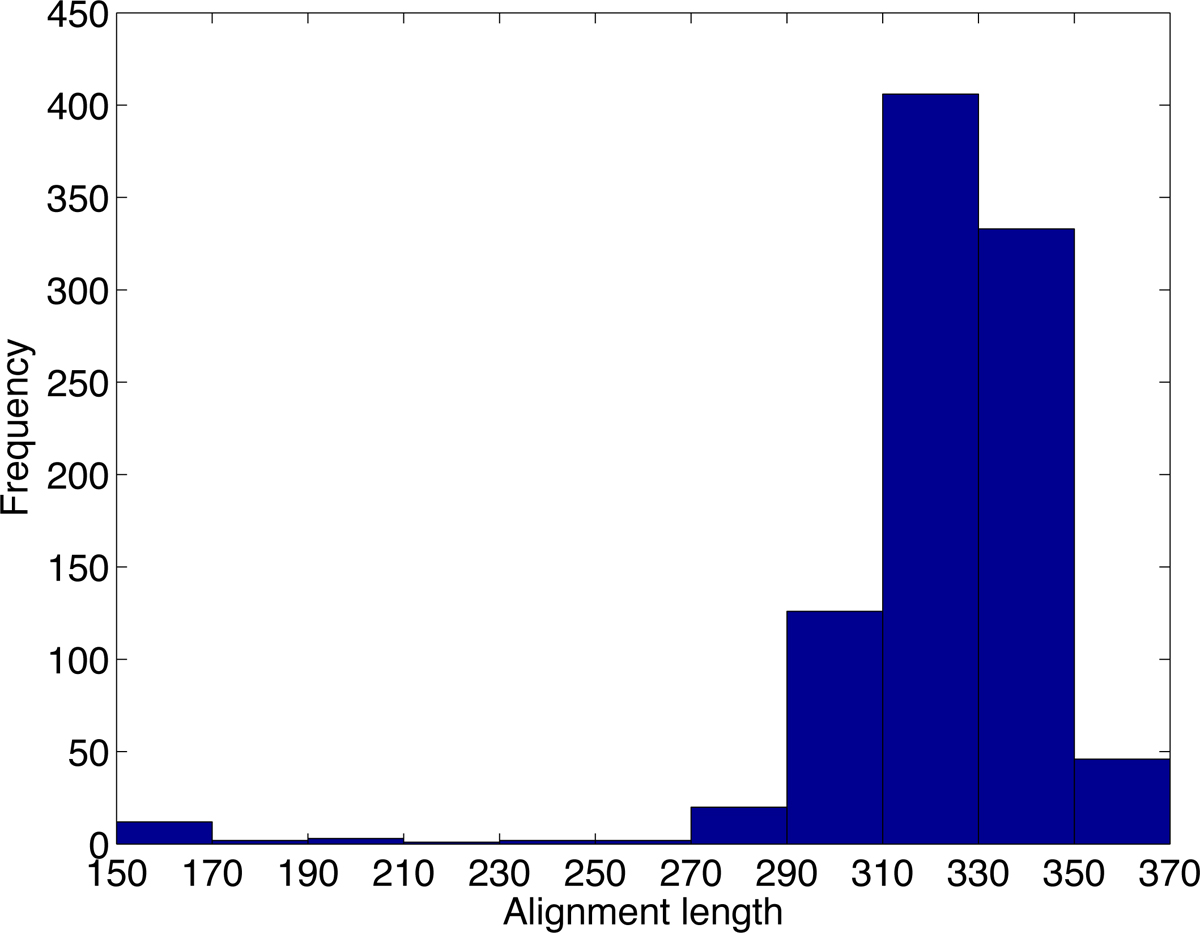
The length distribution of PLAST-ncRNA alignments on chromosome 1.

**Figure 11:**
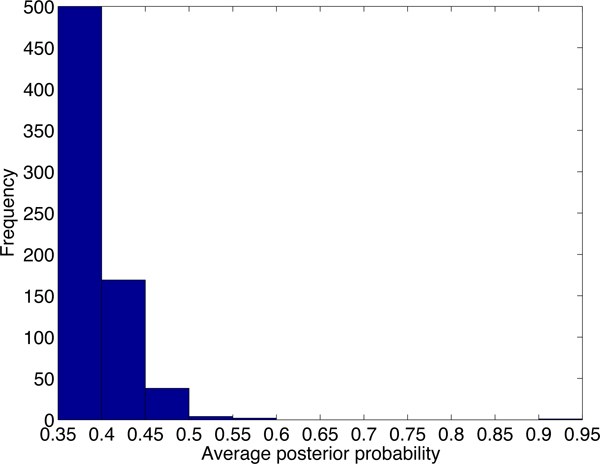
The average posterior probability distribution of PLAST-ncRNA alignments on chromosome 2.

**Figure 12:**
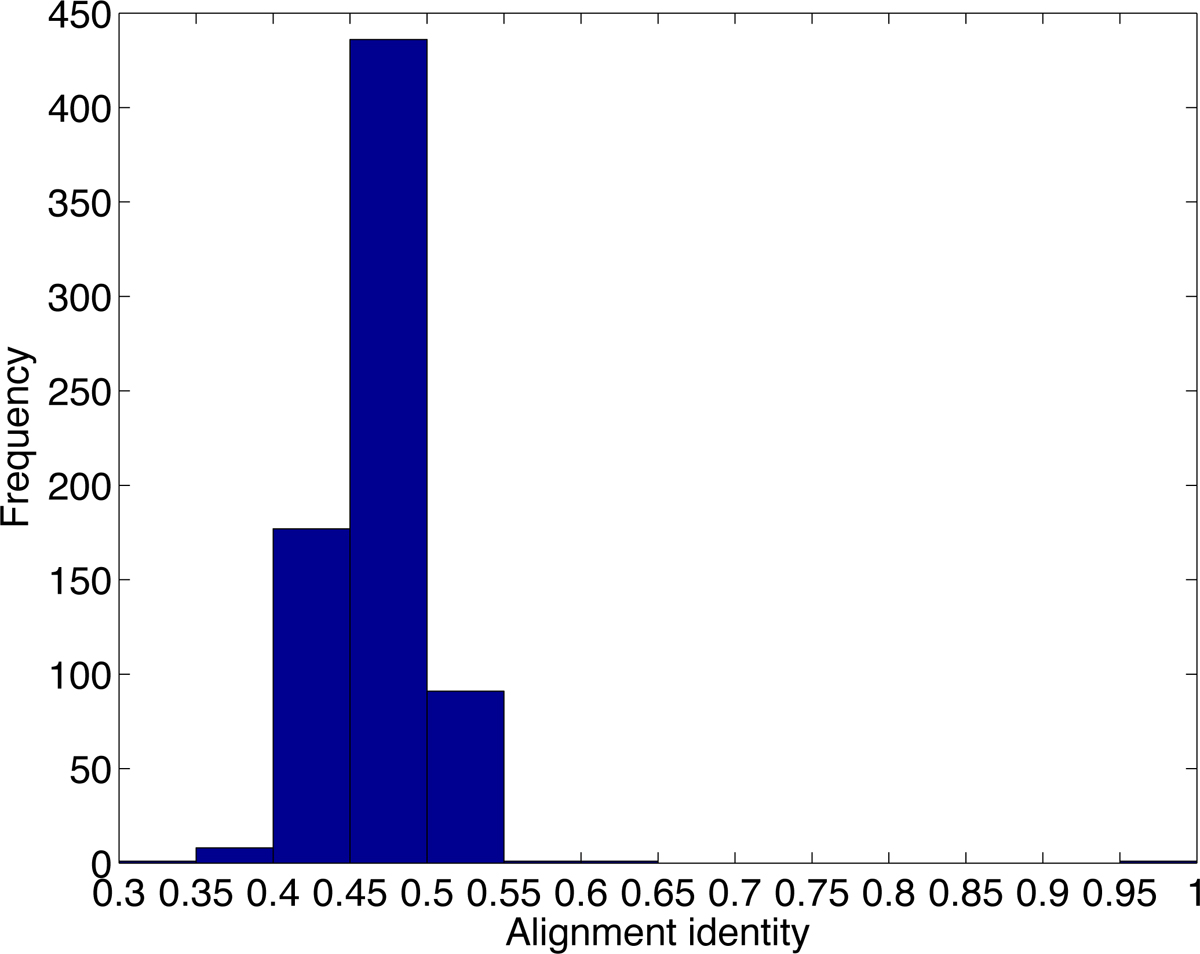
The identity distribution of PLAST-ncRNA alignments on chromosome 2.

**Figure 13:**
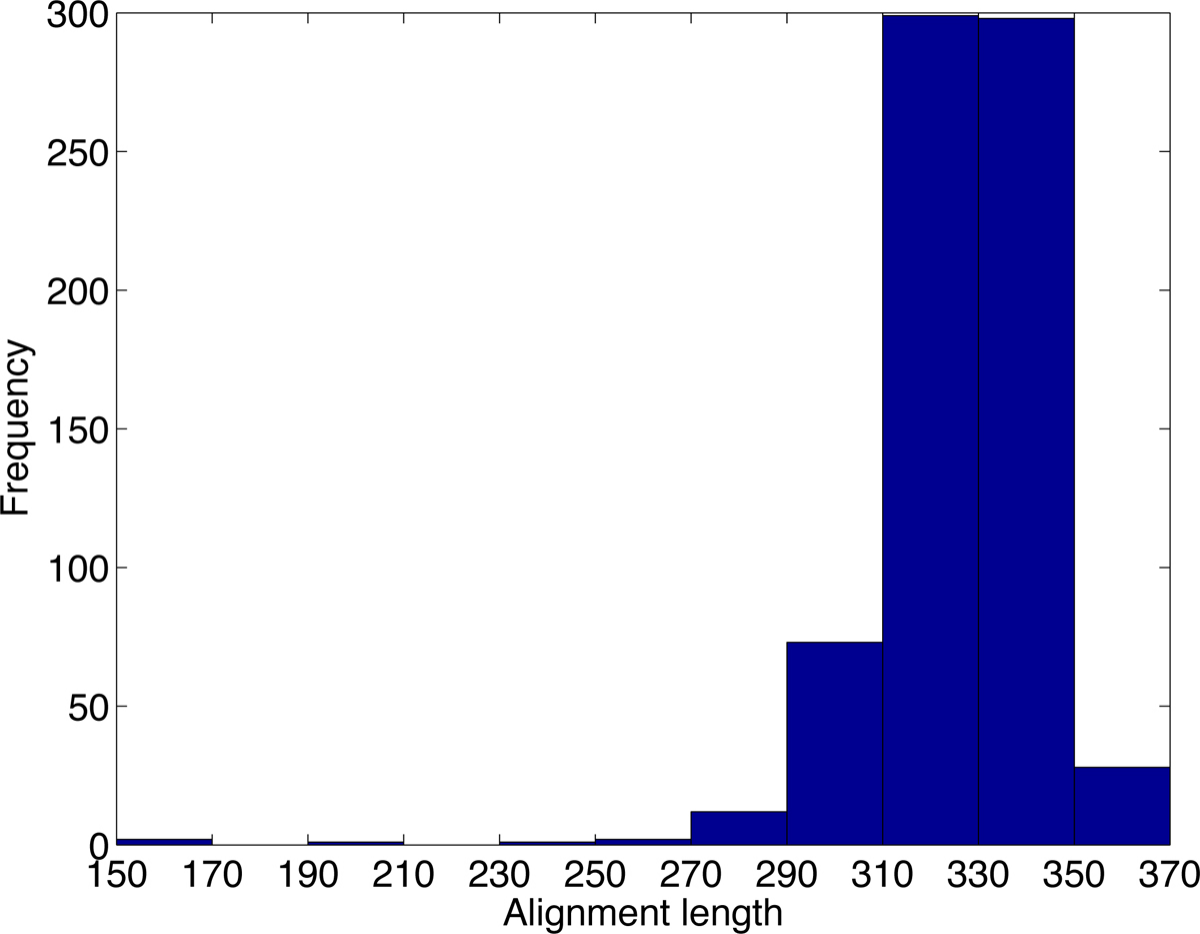
The length distribution of PLAST-ncRNA alignments on chromosome 2.

Note that although the lowest sequence identity allowed by our chosen HD seed *<*200,55*>*is 46%, PLAST-ncRNA is applied to bigger regions around each seed hit. As a local structural alignment, PLAST-ncRNA can report highly structured alignments with very low sequence conservation. This is shown in the identity distribution in Figures 9 and 12. Many of the putative ncRNAs on chromosome 1 are longer than annotated small ncRNAs. This is consistent to previous observation that small ncRNAs tend to have better sequence conservation than long ncRNAs [7].

**Figure 14:**
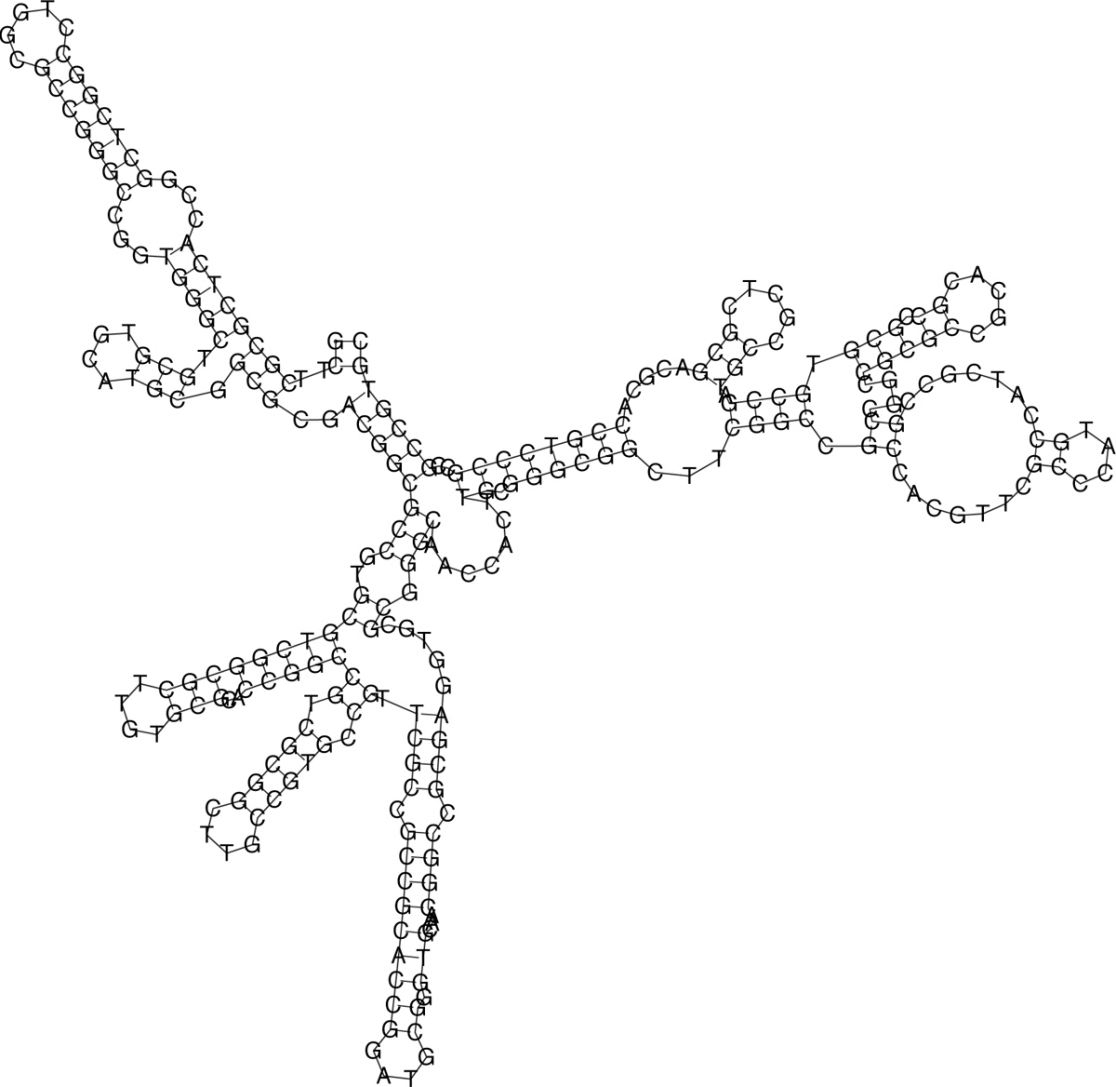
The predicted secondary structure for putative ncRNA 1.

**Figure 15:**
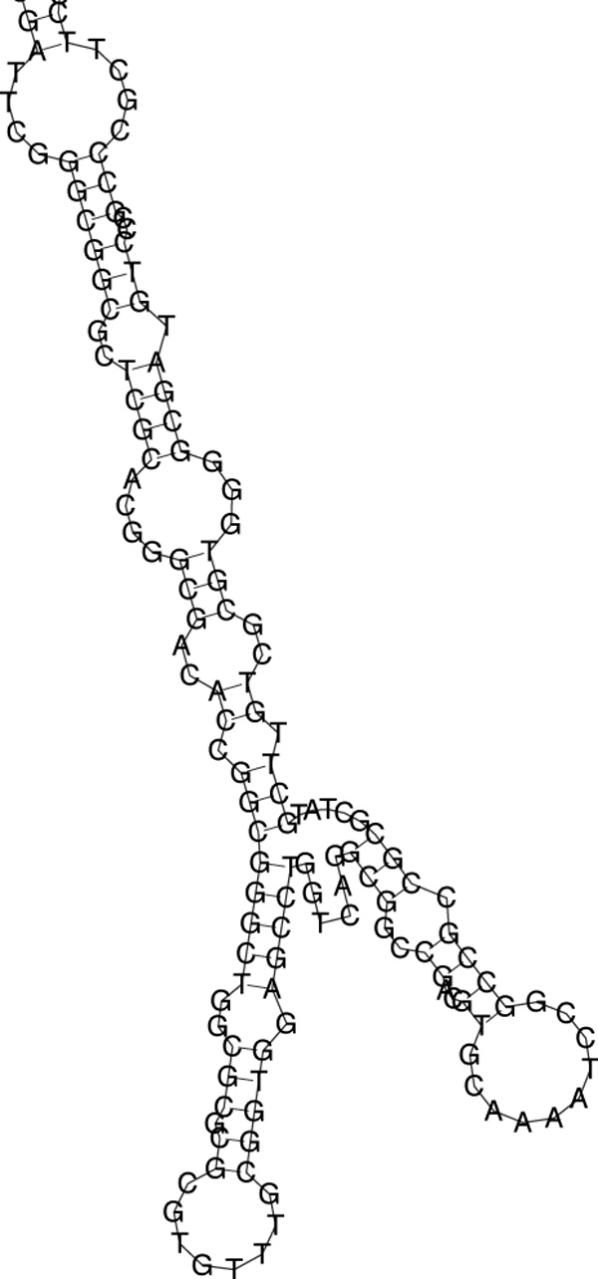
The predicted secondary structure for putative ncRNA 2.

As PLAST-ncRNA does not output the consensus secondary structure, we obtain the structural information from FOLDALIGN. Figures 14 and 15 show the secondary structures of two putative ncRNAs. Their properties including their positions, length, distance to adjacent protein-coding genes etc. are presented in Table 4.

**Table 4:** Properties of two putative ncRNAs on chromosome 1 of J2315

ID	PLAST	FA	Start	End	identity	p-value	5' gene	3' gene	5'	3'
	score	score							D	D
1	0.39	2591	3278580	3278849	0.52	0.022	BCAL2989	BCAL2990	50	53
2	0.38	2538	548365	548654	0.42	0	BCAL0496	BCAL0497	55	267

All of them are conserved in R. solanacearum.

"PLAST" refers to PLAST-ncRNA. "FA" stands for FOLDALIGN. 5' D and 3' D contain the distances to the 5' and 3' neighbor protein-coding genes, respectively.

## Discussion

We applied FOLDALIGN and PLAST-ncRNA as the local alignment tools to regions around HD seed hits. Although both of these tools conduct local alignment, they are based on different rational and have different optimization goals. FOLDALIGN tries to optimize both sequence and structural similarities. PLAST-ncRNA uses posterior probability to conduct sensitive alignment and does not directly incorporate secondary structure information. Yet, we found that the outputs of these two tools share a large overlap. This could indicate that the shared alignments are highly likely to contain true ncRNAs as they achieved high scores using two highly different alignment methodologies. On the other hand, there is a possibility that these two methods tend to have similar false positive hits. Thus, this poses further questions about how to distinguish functional ncRNAs from pseudo-ncRNAs, which can pass the default cutoffs of the alignment tools but lack real functions. Extra evidence beyond high alignment scores is needed. One type of computational evidence is base composition, which can be conveniently incorporated into homology search. Schattner [26] applied base-composition statistics to ncRNA gene finding in a limited number of experiments. It is worth investigating whether these statistics can be applied to different species. Other useful evidence includes the availability of the transcriptomic data, the translation potential, and the genomic context around the local alignments. Finally, if these local alignments can be found in a third related genome, this also provides strong evidence for functional ncRNA search.

In this work, we optimize the HD seeds using all known ncRNAs from different species as the training data. We are aware that different types of ncRNAs share different sequence similarities. For example, tRNA and SECIS are more structural and often share lower sequence conservation than snoRNA and miRNA. If we divide our training set into different groups by average sequence similarities, we will have different optimal seeds for each group. However, there is one difficulty behind this strategy. The sizes of available training data can be quite different for homologous ncRNAs in different groups. For example, there are a large number of snoRNAs and miRNAs in current Rfam database. As their average sequence similarities are high, we will have more training data in that group than other groups. For ncRNAs lacking enough training data, the HD seed design may be highly biased. With the advances of the next-generation sequencing technologies and ncRNA search techniques, we foresee that more and more ncRNAs will be revealed from different species. Enrichment of training data will enable us to design better seeds for ncRNAs with different ranges of sequence similarities in the future.

## Conclusions

Our experimental results show that HD seed matching provides an effective and efficient filtration step for genome-scale ncRNA search. Compared to conventional sequence comparison tools, HD seed matching is more sensitive in identifying ncRNAs with low sequence conservation. By designing a long HD seed, we can control the matching probability to random sequences. Thus, integrating HD seed matching and a sensitive local structural alignment tool provides a complementary ncRNA search method to existing sequence alignment-based implementations. Besides FOLDALIGN and PLAST-ncRNA, other local ncRNA structural alignment tools or classification methods that integrate more features can be applied to examining HD seed hits.

We plan to apply this method to ncRNA identification in available transcriptome datasets. It has been reported that a large portion of transcript reads generated by RNA-seq cannot be mapped to annotated features such as protein-coding genes. It is unknown whether those reads are from functional ncRNAs. Our tool can be used to examine whether the transcribed regions have structural conservation in related genomes when BLAST-like tools fail. We also plan to integrate more biological features to remove hits that are not likely to be ncRNAs.

## Competing interests

The authors declare that they have no competing interests.

## Authors' contributions

YS and AL started this project. YS and OA designed the algorithm. YS designed the experiments and wrote the manuscript. OA implemented the optimal HD seed design. OA carried out the ncRNA search experiments on intergenic regions of human and mouse. OA conducted EVD curve fitting for the FOLDALIGN scores. JL carried out the experiments on the *Burkholderia cenocepacia *J2315 genome.
